# Seasonality Modifies Methylation Profiles in Healthy People

**DOI:** 10.1371/journal.pone.0106846

**Published:** 2014-09-11

**Authors:** Fulvio Ricceri, Morena Trevisan, Valentina Fiano, Chiara Grasso, Francesca Fasanelli, Chiara Scoccianti, Laura De Marco, Anna Gillio Tos, Paolo Vineis, Carlotta Sacerdote

**Affiliations:** 1 Unit of Cancer Epidemiology – CERMS, Department of Medical Sciences, University of Turin and Città della Salute e della Scienza Hospital, Turin, Italy; 2 Department of Mathematics, University of Turin, Turin, Italy; 3 IARC, Lyon, France; 4 Human Genetics Foundation (HUGEF), Turin, Italy; 5 Imperial College, London, United Kingdom; University of Navarra, Spain

## Abstract

DNA methylation is a well-characterized epigenetic modification that plays an important role in the regulation of gene expression. There is growing evidence on the involvement of epigenetic mechanisms in disease onset, including cancer. Environmental factors seem to induce changes in DNA methylation affecting human health. However, little is known about basal methylation levels in healthy people and about the correlation between environmental factors and different methylation profiles. We investigated the effect of seasonality on basal methylation by testing methylation levels in the long interspersed nucleotide element-1 (LINE-1) and in two cancer-related genes (*RASSF1A* and *MGMT*) of 88 healthy male heavy smokers involved in an Italian randomized study; at enrolment the subjects donated a blood sample collected in different months. Methylation analyses were performed by pyrosequencing. Mean methylation percentage was higher in spring and summer for the LINE1, *RASSF1A* and *MGMT* genes (68.26%, 2.35%, and 9.52% respectively) compared with autumn and winter (67.43%, 2.17%, and 8.60% respectively). In particular, LINE-1 was significantly hypomethylated (p = 0.04 or 0.05 depending on the CpG island involved) in autumn and winter compared with spring and summer. Seasonality seems to be a modifier of methylation levels and this observation should be taken into account in future analyses.

## Introduction

DNA methylation is a well-characterized epigenetic modification that involves the addition of a methyl group to cytosine when paired to guanine (CpG). DNA methylation plays an important role in the regulation of gene expression. Alterations in DNA methylation pattern mostly lead to silencing of inducible genes (promoter hypermethylation) or to re-expression of physiologically silenced repetitive sequences and transposable elements (hypomethylation). As a result, molecular pathway deregulation as well as chromosomal instability and increased mutational events may occur, thus favouring onset of diseases, including cancer [1]. Environmental factors seem to affect DNA methylation patterns. [2] Exposure to carcinogens [3]–[5], air pollution [6], [7], and nutritional factors [8] were described to be associated with variation of DNA methylation status in exposed subjects compared to unexposed ones.

Little is known about modulation in the basal methylation level in healthy people and in particular about the correlation between season-linked environmental factors and different methylation profiles, although seasonality was described to sporadically influence epigenetic events in different organisms, including plants [9], [10], animals [11], and humans [12]–[14].

To investigate in depth if seasonality may impact methylation level in healthy people, we focused on selected genes frequently tested in studies of association between DNA methylation and cancer. We aimed at evaluating if variability in healthy subjects' basal methylation level has to be taken into account to avoid biased results. Three genes were selected to be investigated in the blood DNA of a series of healthy people enrolled in a previous study of association between diet and DNA damage [15]: LINE-1, *RASSF1A* and *MGMT*. Transposable methylation of LINE-1 elements (long interspersed nucleotide element-1) is generalized to reflect global DNA methylation [16], [17]. *RASSF1A* (*Ras-association domain family 1 isoform A*) is a tumour suppressor gene regulated by promoter methylation, which leads to inhibition of its expression. Promoter hypermethylation of this gene is related to carcinogenesis. [18]–[20] *MGMT* (*O6-methylguanine DNA methyltransferase*) is a gene involved in DNA repair mechanisms. The promoter hypermethylation of *MGMT* inactivates different DNA repair pathways and as suggested [21], [22], this event occurs in sporadic and hereditary cancer.

## Subjects and Methods

### Subjects

In the frame of an Italian randomized trial (approved by the local ethics committee) focused on the study of the association between diet and DNA damage in heavy smokers (for further details see ref [15], [23], [24]); hence 88 healthy heavy smokers of air-cured tobacco were recruited among Italian blood donors. Participants were all males, aged 35–70, residents in Turin metropolitan area (Northern Italy). At the baseline, volunteers filled in a validated food frequency questionnaire. [25] Twenty mL of non-fasting blood were collected at the beginning of the study and one year later; after processing, they were fractioned in plasma and buffy coat and immediately stored in aliquots at −20. Herein, only samples collected at time zero were used. Because the study subjects were enrolled at different months of the year, we could group the blood samples by season.

### Ethics statement

All participants signed an informed consent form. The study was approved by the ethical committee of the Department of Biomedical Sciences and Human Oncology, University of Torino, and was monitored by the Association of General Practitioners of the Province of Torino.

### DNA extraction and sodium bisulfite treatment

Genomic DNA was extracted from 200 µl aliquots of buffy coat through QIAamp DNA Blood Mini Kit (Qiagen, Hilden, Germany) according to manufacturer's instructions, with a final elution in 70 µl of TE elution buffer.

All the genomic DNA samples, in addition to positive synthetic controls for methylated and unmethylated status, underwent bisulfite modification using Epitect Bisulfite Kit (Qiagen, Hilden, Germany) according to manufacturer's instructions.

### Methylation analysis

Pyrosequencing assays were performed on a PyroMark Q24 using PyroMark Gold reagents (Qiagen). This updated system incorporates a dedicate software for CpG analysis which allows a more accurate quantization of methylation compared with AQ software of PyroMark Q96 that is still being widely used for this purpose.

Primers were generated according to PyroMark Assay Design software (Qiagen) for all the gene specific target sequences.

Preliminary PCR targeting the selected gene sequences to be investigated were performed by using primers listed in Table 1 at corresponding annealing temperature.

**Table 1:** Pyrosequencing primers and annealing temperature.

GENE	Forward	Reverse	Sequencing	Annealing T
MGMT*	GTTTTAGGAGGGGAGAGATT	CCTTAATTTACCAAATAACCC-biot	GGGATTTTTATTAAG	59°C
RASSF1A§	AGTTTGGATTTTGGGGGAGG	CAACTCAATAAACTCAAACTCCCC-biot	GGGTTAGTTTTGTGGTTT	59°C
LINE-1#	biot-TAGGGAGTGTTAGATAGTGG	AACTCCCTAACCCCTTAC	AACTCCCTAACCCCTTAC	55°C

* Target: 194 bp of MGMT (Gene ID 4255 at position 44526–44719) promoter sequence including 6 CpGs at position: 44600, 44604, 44607, 44614, 44621, 44623.

§ Target: 136 bp of RASSF1A (Gene ID 11186 at position 1697–1833) promoter sequence including 6 CpGs at position: 1746, 1750, 1755, 1757, 1767, 1780.

# Target: 108 bp of LINE-1 (GeneBank X58075.1 at position 117–224) promoter sequence including 6 CpGs at position: 156, 131, 165, 167, 172, 182.

The preliminary PCR reactions were performed in a total volume of 35 µl containing buffer (KCl) 1X, MgCl_2_ 2 mM, dNTPs 0.8 mM, 0.5 µM each primer Taq polymerase 1 U and 3 µl converted DNA with the following touchdown PCR profile decreased of 3°C every 3 cycles until the specific annealing temperature was reached: 94°C for 5 min followed by 3 cycles at 94°C for 30 sec, starting annealing temperature at 62°C for 30 sec, 72°C for 30 sec. When the gene specific annealing temperature was reached, further 30 cycles were performed. Amplicons were analyzed by gel electrophoresis on a 2% agarose gel stained with ethidium bromide and visualized by ultraviolet trans-illumination. Twenty µl of PCR product were added to 18 µl of distilled water and incubated under shaking with 40 µl of binding buffer pH 7.6 (10 mM Tris-HCl; 2 M NaCl; 1 mM EDTA; 0.1% Tween 20) and 2 µl sepharose beads covered with streptavidin. PCR products were washed with ethanol 70%, denatured with NaOH 0.2 M and re-washed with Tris-Acetate 10 mM pH 7.6. Pyrosequencing reaction was performed in 25 µl of annealing buffer [23.25 µl of 20 mM Tris-Acetate+5 mM MgAc_2_, 0.15 µl of sequencing primer (0.3 µM) and 1.25 µl of dimethyl sulfoxide (DMSO)]. Assays were created according to manufacturer's instruction and the nucleotide dispensation order was suggested by the software used.

For consistency we tested the three genes by performing all the methylation analyses on the PyroMark Q24, even though *RASSF1A* and LINE-1 had been already analyzed in a previous study. [26] Use of different pyrosequencing instruments incorporating different analytical software may explain some discrepancies in the absolute values of methylation percentage obtained.

### Statistical analysis

We presented our results according to seasonality. Two groups, one including samples collected in spring and summer and another consisting of samples collected in autumn and winter, were compared.

We presented summary data as means, medians and standard deviations (SD) for continuous variables and as percentages for categorical variables. Covariates/potential confounders were tested using Wilcoxon rank sum test or Chi-square test, for continuous variables or categorical variables, respectively. In order to test differences in methylation levels we used Wilcoxon rank sum test with continuity correction. We also presented the differences in methylation levels for each tested CpG site. To explain the seasonal differences, an analysis on the intake of nutrients eaten the day before sampling was conducted. Correlations between food groups and methylation were tested using Pearson's correlation coefficient.

All tests were two-sided and we used a 5% significance threshold.

Analyses were performed using SAS V9.3.

## Results

The subjects' mean age involved in the study was 52.42 (6.74 SD) and they were all males and heavy smokers at the time of the analysis. The mean of Body Mass Index (BMI) was 26.40 kg/m^2^ (3.29 SD). Table 2 shows that no differences were found in the study for covariates/potential confounders in different seasons.

**Table 2:** Descriptive analysis of potential confounders by seasonality.

Study subjects N = 88	Spring/Summer N = 29 mean% (SD)	Autumn/Winter N = 59 mean% (SD)	P-values
Age	51.19 (7.02)	53.03 (6.57)	0.30
BMI	26.94 (3.88)	26.13 (2.96)	0.18

P – values are based on Wilcoxon rank sum test or chi square test.

**Figure 1:**
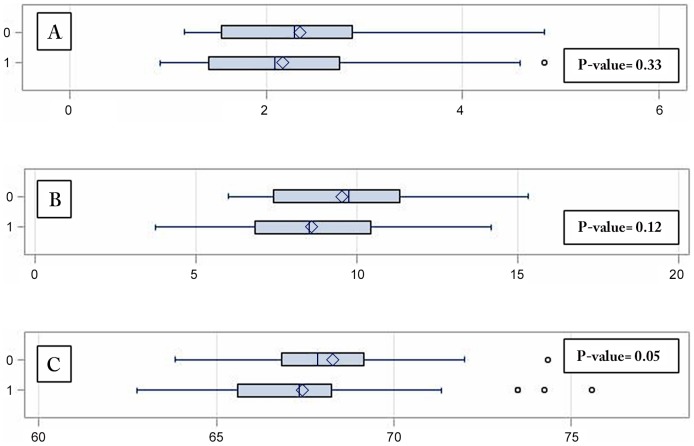
Box plot for percentage of methylation of *RASSF1A* (Panel A), *MGMT* (Panel B), LINE1 (Panel C) divided by seasonality (0 =  autumn/winter; 1 =  spring/summer). P-values are based on Wilcoxcon rank sum test.

Results of the overall methylation analysis are shown in Table 3 where methylation is reported as mean/median percentage of methylation of all the CpG sites investigated for the specific gene and in Figure 1, in which average methylation for the different CpG islands is reported for each gene. In all the loci analyzed, the methylation status was higher in spring and summer compared with autumn and winter. In particular, LINE-1 was borderline significantly hypermethylated (p = 0.05) in spring/summer compared with autumn/winter and this difference is significant for 3 over 6 sites (CpG II, III, VI) in LINE-1 and for 1 site (CpG II) in *MGMT*. The samples of the paired season group showed similar methylation levels (data not shown).

**Table 3:** Prevalence of methylation (mean/median percentage of methylation) in all the 18 CpG islands analized.

CpG Island	Spring/Summer		Autumn/Winter		p-value
	Mean	Median	Mean	Median	
**LINE1_CpG_I**	62.55	62.00	62.03	62.00	0.34
**LINE1_CpG_II**	76.24	74.00	74.00	73.00	0.05
**LINE1_CpG_III**	52.55	53.00	51.51	52.00	0.05
**LINE1_CpG_IV**	59.55	60.00	58.92	59.00	0.12
**LINE1_CpG_V**	91.66	91.00	91.31	90.00	0.24
**LINE1_CpG_VI**	67.62	68.00	66.76	67.00	0.04
**MGMT_CpG_I**	20.19	20.00	18.48	18.50	0.15
**MGMT_CpG_II**	12.22	12.00	10.39	10.00	0.03
**MGMT_CpG_III**	8.78	9.00	7.96	7.50	0.19
**MGMT_CpG_IV**	3.74	4.00	3.48	3.00	0.34
**MGMT_CpG_V**	7.30	7.00	6.69	6.00	0.21
**MGMT_CpG_VI**	5.26	5.00	4.52	5.00	0.08
**RASSF_CpG_I**	1.61	1.50	1.38	1.00	0.15
**RASSF_CpG_II**	3.54	4.00	3.12	3.00	0.16
**RASSF_CpG_III**	3.61	3.50	3.21	3.00	0.13
**RASSF_CpG_IV**	1.43	1.00	1.40	1.00	0.72
**RASSF_CpG_V**	1.64	1.00	1.47	1.00	0.23
**RASSF_CpG_VI**	2.57	2.50	2.28	2.00	0.27

P-values are based on Wilcoxcon rank sum test.

The additional analysis on nutrients showed a slight and non-significant difference for Vitamin B6 (1.45 mg in autumn/winter vs 1.52 in spring/summer), B9 (195 µg vs 206 µg), and B12 (0.97 mg vs 1.00 mg), while a significant increase in autumn/winter vs. spring/summer was found for flavonoids (107.79 mg vs 65.69 mg, p-value = 0.03).

Only low correlations (r<|0.30|) between methylation and food groups were found, although some of correlation coefficients were statistically significant (data not shown).

## Discussion

There is growing evidence on the involvement of epigenetic mechanisms in disease onset, in particular cancer. However, little is known about the determinants of basal methylation levels in healthy people. Whereas studies in healthy people were mainly focused on the identification of a correlation between methylation and gene expression [27], few reports are available on intra- and inter-individual differences in methylation among healthy people. Commonly, healthy people are included in case-control studies and sources of variability in methylation levels of the control group subjects are rarely considered.

In our study we found differences in methylation levels of selected genes in healthy adults according to seasonality. We divided the methylation results into two groups showing overall similar intra-group methylation levels: one including subjects enrolled in spring and summer and the other including those enrolled in autumn and winter. This seasonal grouping is in line with previous reported data on seasonality [14] and allowed us to compare groups with a larger sample size.

In the spring and summer group we observed a pattern of higher methylation in all the promoter regions of genes we analyzed, compared with the other. The differences, in spite of not being statistically significant (likely due to the small sample size of the study), but borderline for LINE-1, are suggestive of the possible impact on basal DNA methylation levels of seasonal determinants. The observed differences are not likely to reflect exposure to inhaled tobacco carcinogens which did not vary between seasons. Also, while the study subjects are slightly overweight on average (Table 2), at present there is no evidence of an association of DNA methylation with body mass index. [28] Air pollution could be one of the likely determinants: Baccarelli et al. [7] showed that variation in methylation levels of LINE-1 in healthy subjects was not only associated with season, but also with the day of the week, being higher on Wednesdays than Mondays.

Our data are in line with recent reports showing evidence of seasonal variability in air composition, daytime light, temperature and habits that may affect DNA methylation in all the living organism, human, animals and plants.

In several studies [29]–[31] short-term effects of pollutants on health were more evident in spring and summer than in the coldest seasons, and more in southern countries than in northern countries. One proposed interpretation of this finding has been that higher exposure to outdoor air pollutants during the summer period might be due to increased indoor ventilation and seasonal differences in outdoor human activity. Other differences may be due to the chemical mixture in outdoor pollution that varies with season, source pattern and weather. [31]

Additionally, ozone levels have an opposite seasonality pattern compared with other air pollutants; the highest levels of ozone in the Northern Hemisphere were found in the spring and summer and the lowest in the autumn and winter. Ozone has been shown to have important detrimental health effects [32] and it is known that some individuals are particularly susceptible to ozone adverse effects. Genetic and epigenetic mechanisms have been invoked to explain differential susceptibility and alterations in DNA methylation could be involved.

Circadian rhythms were also described to affect basal DNA methylation status. [33]–[35] Exposure to light at night through long-term shift work can influence DNA methylation and may result in epigenetic alterations of biological pathways and cancer-related genes.

Another possible explanation of the association between variation in healthy people's basal methylation levels and seasonality is the change in outdoor temperature. Seasonal environmental temperature changes regulate the life of plants and aquatic species. Adaptation to cold and warm conditions requires dramatic changes in gene expression. For example, in eurythermal fish adaptation to cold conditions requires considerable changes at cellular level in gene expression regulated by epigenetic mechanisms, such as methylation. [11] According to Baccarelli [7] LINE1 methylation showed an association with mean outdoor temperature of the day when the blood was drawn; despite not being significant, this factor was consistent with changes observed by season.

We observed a similar relationship between biomarker and seasonality in a pooled analysis on bulky DNA adducts. [36]. DNA adducts showed lower levels in spring than in winter. For this result our explanation was more straightforward, i.e. a protective effect of seasonal intakes of fresh fruit and vegetables and higher levels of exposure to particulate outdoor pollution in winter.

Results from the European Prospective Investigation into Cancer and Nutrition (EPIC study) [37] showed that dietary folate intake is lower in summer, especially in southern European countries, because summer heat causes bolting and foliage deterioration in vegetables. Moreover, in a recent paper [38] the authors observed that naturally occurring seasonal variations in food consumption patterns have a profound effect on methyl-donor biomarker status. Therefore, despite expecting lower methylation levels in summer, we found an opposite result, possibly suggesting that dietary intake of folate may not be major determinant of methylation levels. [26]

Similar evidence was highlighted for the increased level of flavonoids in autumn/winter. [26] Although the debate is still open [39], different *in vitro* studies showed that flavonoids (in particular, genistein) act as a DNA demethylating agent, inducing a dose-dependent inhibition of the DNA-methyltrasferase activity. [40]–[42]

It is also to be taken into account that in different seasons can occur a preferential selection of particular peripheral blood cell types [43]. Thus white blood cell may have different patterns of DNA methylation.among seasons.

Finally, many seasonally related candidate gene pathways seem to be regulated by epigenetic mechanisms, DNA methylation included. This evidence is strengthened by the results described in rodent species, where deeper laboratory investigations can be performed. [44] For example, in blood of hibernating chipmunks, the hibernation-specific protein-27 is known to be upregulated by DNA methylation. [45] As well, in ground squirrels has been shown that a protein linked to the regulation of the circadian rhythms in the liver affected the de novo methyltransferase probably resulting in the metabolic depression during hibernation. [46] In mice, DNA methylation has been shown to play a role in the suprachiasmatic nucleus of the hypothalamus, which regulates circadian behaviour in mammals. [47]

Moreover, epigenetic changes may explain the seasonal reproductive phenotypes in seasonally breeding vertebrates. In hamsters, changes in photoperiod have been shown to induce variation in reproductive phenotypes mediated by DNA methylation. [48]

One limitation of our study is that the differences we found in methylation levels by seasonality were small, but this is consistent with variability of the basal methylation level, specifically in cancer-related genes such as *RASSF1A* and *MGMT* which are a priori expected to show small changes in promoter methylation in healthy people. Otherwise, LINE-1 sequences are expected to be highly methylated since they are physiologically silenced in healthy people. Because these sequences are highly repetitive along the genome, even hardly detectable differences could assume relevance in terms of chromosomal instability or increased mutational events.

Another limitation is the study was performed only on male heavy-smoker, hence further investigations are needed to extrapolate our findings to more general population.

However the strength of this study is represented by investigating for the first time in healthy subjects a seasonal pattern of DNA methylation in genes usually studied in association with neoplastic diseases.

In conclusion, seasonality seems to be a modifier of basal DNA methylation level in healthy people. These findings suggest that variability in the basal methylation level of healthy controls should be taken into account in future methylation data analyses. Adjustment for season of sample collection is advisable to avoid biased results, specifically when the difference in methylation levels of the study groups is expected to be moderate.
